# Epilepsy. Atropine

**Published:** 1854-12

**Authors:** 


Epilepsy. Atropine.—Dr. Lange has used atropine in ten cases of epilepsy (three men and seven women). The three men, who had suffered from the disease for many years, were cured in three, five, and six weeks. Two of the women were not improved, one died, and three appeared to be cured, as, after from five to eleven months, they had had no fresh attacks. In the last case, one of epilepsy and commencing idiotcy, the atropine failed. The dose appears to have been about the 1-100th of a grain. M. Delasiauve, in his late treatise on "Epilepsy" states that he has experimented with belladonna for many years at the Bicêtre, and that, while he has seen some cases in which the fits were for the time suspended, he has only seen one instance of cure.—*Schmidt's Jahrb.*

